# Activation of volume-sensitive outwardly rectifying chloride channel by ROS contributes to ER stress and cardiac contractile dysfunction: involvement of CHOP through Wnt

**DOI:** 10.1038/cddis.2014.479

**Published:** 2014-11-20

**Authors:** M Shen, L Wang, B Wang, T Wang, G Yang, L Shen, T Wang, X Guo, Y Liu, Y Xia, L Jia, X Wang

**Affiliations:** 1 Department of Geriatrics, Xijing Hospital, Fourth Military Medical University, Xi'an, China; 2 Department of Cardiology, Hainan Branch of PLA General Hospital, Sanya, China; 3 Department of Biochemistry and Molecular Biology, Fourth Military Medical University, Xi'an, China

## Abstract

Endoplasmic reticulum (ER) stress occurring in stringent conditions is critically involved in cardiomyocytes apoptosis and cardiac contractile dysfunction (CCD). However, the molecular machinery that mediates cardiac ER stress and subsequent cell death remains to be fully deciphered, which will hopefully provide novel therapeutic targets for these disorders. Here, we establish tunicamycin-induced model of cardiomyocyte ER stress, which effectively mimicks pathological stimuli to trigger CCD. Tunicamycin activates volume-sensitive outward rectifying Cl^−^ currents. Blockade of the volume-sensitive outwardly rectifying (VSOR) Cl^−^ channel by 4,4'-diisothiocya-natostilbene-2,2'-disulfonic acid (DIDS), a non-selective Cl^−^ channel blocker, and 4-(2-butyl-6,7-dichlor-2-cyclopentyl-indan-1-on-5-yl) oxybutyric acid (DCPIB), a selective VSOR Cl^−^ channel blocker, improves cardiac contractility, which correlates with suppressed ER stress through inhibiting the canonical GRP78/eIF2*α*/ATF4 and XBP1 pathways, and promotes survival of cardiomyocytes by inverting tunicamycin-induced decrease of Wnt through the CHOP pathway. VSOR activation of tunicamycin-treated cardiomyocytes is attributed to increased intracellular levels of reactive oxygen species (ROS). Our study demonstrates a pivotal role of ROS/VSOR in mediating ER stress and functional impairment of cardiomyocytes via the CHOP-Wnt pathway, and suggests the therapeutic values of VSOR Cl^−^ channel blockers against ER stress-associated cardiac anomalies.

The endoplasmic reticulum (ER) is characterized as an organelle that participates in the folding of membrane and secretory proteins.^1,2^ Efficient functioning of the endoplasmic reticulum is important for cell function and survival. Perturbations of ER homeostasis by energy deprivation and glucose,^3^ viral infections^4^ and accumulation of misfolded and/or unfolded proteins^2^ interfere with ER function, leading to a state of ER stress.^5, 6, 7^ A cohort of chemicals, for example, tunicamycin and thapsigargin, also trigger ER stress.^8, 9, 10^ Thapsigargin disrupts the calcium storage of ER by blocking calcium reuptake into the ER lumen, thus by depleting calcium from the organelle.^11^ In particular, tunicamycin is a highly specific ER stress inducer by inhibiting *N*-linked glycosylation of protein, representing a well-documented method to artificially elicit unfolded protein response.^8^ In response to ER stress, ER chaperones such as glucose-regulated protein 78 kDa (GRP78) and glucose-regulated protein 94 kDa (GRP94) are upregulated to facilitate the recovery of unfolded or misfolded proteins.^12^ ER stress may act as a defense mechanism against external insults; however, prolonged and/or severe ER stress may ultimately trigger apoptosis.^8^ The C/EBP homologous protein (CHOP) has been defined as a pivotal mediator of cell death signaling in ER stress.^13, 14^ Accumulating evidence has demonstrated that ER stress-induced cell death is an essential step in the pathogenesis of a wide variety of cardiovascular diseases such as ischemia reperfusion heart diseases,^15^ atherosclerosis,^5, 16, 17, 18^ myocardial infarction,^19^ hypertension^20, 21^ and heart failure.^8, 22, 23^ Inhibiting ER stress has great therapeutic values for cardiac anomalies. However, the precise mechanism involved in ER stress-induced cardiovascular diseases has not been well identified, which impedes the translation of our understanding of ER stress-induced cardiovascular anomalies into effective therapeutic strategies. Apoptosis induction requires persistent cell shrinkage, named apoptotic volume decrease (AVD).^24, 25, 26, 27^ It is an early prerequisite for the activation of caspases.^24^ In various types of cells including cardiomyocytes, AVD process is accomplished by the activation of volume-sensitive outwardly rectifying (VSOR) Cl^−^ channel and is concomitant with the egress of water from the cells undergoing mitochondrion-initiated or death receptor-induced apoptosis.^25, 28, 29, 30^ Although inhibition of VSOR Cl^−^ channel by DIDS (4,4'-diisothiocyanatostilbene-2,2'-disulphonic acid) and DCPIB (4-(2-butyl-6,7- dichlor-2-cyclopentyl-indan-1-on-5-yl) oxybutyric acid) blocked AVD and rescued cardiomyocytes from mitochondrial and death receptor pathway-induced apoptosis,^31, 32^ it remains largely unknown concerning the role of VSOR Cl^−^ channel and how it is regulated in ER stress-induced apoptotic cardiomyocyte death.

Emerging evidence indicates that Wnt signal pathways are found to be anti-apoptotic in the cardiovascular diseases,^33, 34, 35^ regulating crucial aspects of cardiovascular biology. However, up to now, its activity in ER stress-induced apoptosis and in the process of AVD in cardiomyocytes remains elusive.

In the present study, we probed the role of VSOR Cl^−^ channel in ER stress-induced apoptosis of cardiomyocytes, which intimately correlates with cardiac contractile dysfunction (CCD). We hypothesized that VSOR Cl^−^ channel controls the process of AVD occurring concomitantly with ER stress-induced apoptosis of cardiomyocytes. To test this hypothesis, we investigated VSOR Cl^−^ currents in cardiomyocytes treated with the ER stress inducer tunicamycin. The pathophysiological role of VSOR Cl^−^ channel and the potential signaling mechanisms in the development of ER stress-induced apoptosis in CCD were also dissected.

## Results

### ER stress inducer tunicamycin rapidly activates volume-sensitive chloride currents in cardiomyocytes

**Figure 1:**
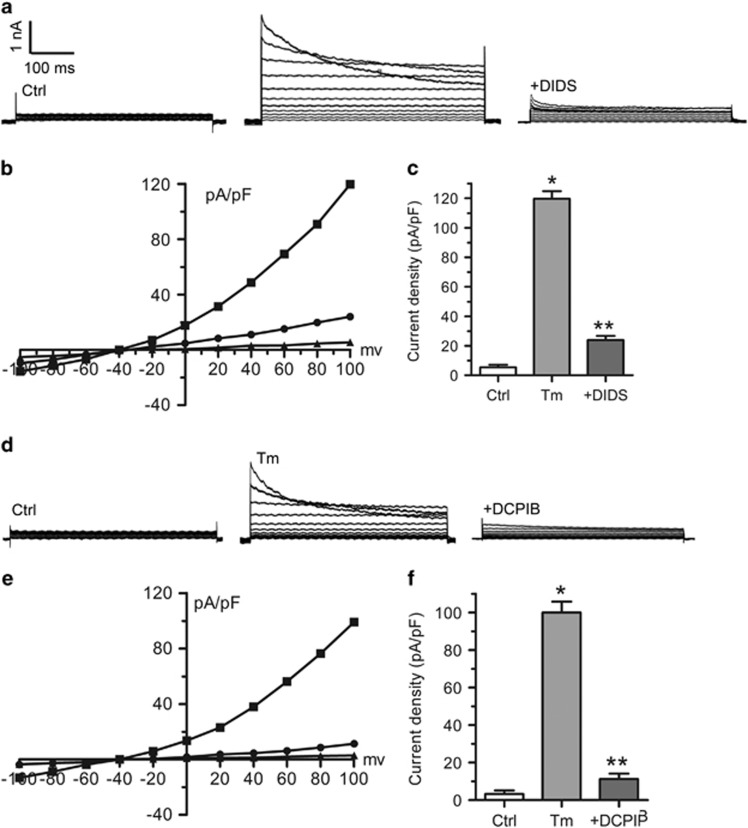
Increased VSOR Cl^−^ currents in tunicamycin exposed cardiomyocytes. (**a**) Negligible background Cl^−^ currents recorded under isosmotic solution (Ctrl). Tm (3 *μ*g/ml)-induced Cl^−^ currents exhibiting representative properties of VSOR Cl^−^ currents (Tm). Tm-induced VSOR Cl^−^ currents were inhibited by adding DIDS (500 *μ*M); *n*=5 for each group. (**b**) Corresponding current-voltage (I-V) relationship for the mean current densities of Ctrl (▴), Tm (▪) and Tm with DIDS (●) conditions. (**c**) Current densities at +100 mV from **b**. *P<0.05 *versus* Ctrl; **P<0.05 *versus* Tm, *n*=5. (**d**) Negligible background Cl^−^ currents recorded under isosmotic solution (Ctrl). Tm (3 *μ*g/ml)-induced Cl^−^ currents exhibiting representative properties of VSOR Cl^−^ currents (Tm). Tm-induced VSOR Cl^−^ currents were inhibited by adding DCPIB (10 *μ*M); *n*=5 for each group. (**e**) Corresponding current-voltage (I-V) relationship for the mean current densities of Ctrl (▴), Tm (▪) and Tm with DCPIB (●) conditions. (**f**) Current densities at +100 mV from (**e**). *P<0.05 *versus* Ctrl; **P<0.05 *versus* Tm, *n*=5

Volume-sensitive chloride channel is critically involved in apoptotic volume decrease (AVD) and cell death of the cardiovascular diseases. Tunicamycin has been commonly employed as a classical pro-apoptotic inducer through the ER stress pathway.^10^ To explore whether volume-sensitive chloride channel also participates in ER stress-induced apoptosis, we examined the effect of ER stress inducer tunicamycin on VSOR Cl^−^ currents in cardiomyocytes. The cellular electro-physiological studies were performed to directly monitor the VSOR Cl^−^ currents. Cardiomyocytes were stimulated with tunicamycin (3 *μ*g/ml, 10–15 min), and the properties of whole cell currents were recorded. Compared with the currents recorded under control conditions, tunicamycin challenges rapidly increased the conductance, which was likely to be I_cell,swell_: voltage-dependent inactivation at large positive voltages (Figure 1a) and outward rectification (Figure 1b), Furthermore, the tunicamycin-induced currents were markedly inhibited by VSOR blockers DIDS (Figures 1a, 500 *μ*M, 5–6 min, 82.05±4.37%, *n*=5, *P*<0.05) and DCPIB (Figures 1d, 10 *μ*M, 6–7 min, 91.33±5.57%, *n*=5, *P*<0.05). The properties of tunicamycin-induced current changes indicate that tunicamycin activates VSOR Cl^−^ currents in cardiomyocytes.

### VSOR Cl^−^ channel blockers rescue ER stress and cell injury in tunicamycin-exposed cardiomyocytes

**Figure 2:**
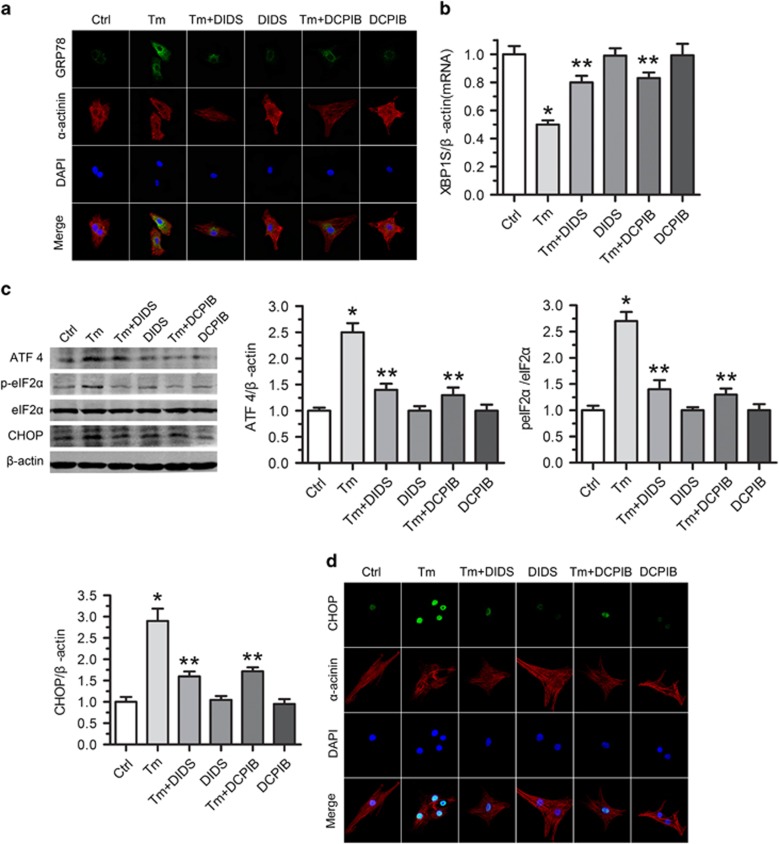
VSOR Cl^−^ channel blockers rescue tunicamycin-induced ER stress in cardiomyocytes. Cardiomyocytes were treated with Tm (100 ng/ml) in the presence or absence of DIDS or DCPIB for 24 h. (**a**) Representative images of immunostaining for GRP78 (Green). Nuclei were labeled with DAPI; *n*=5 for each group. (**b**) qRT-PCR assay for XBP1S expression. *β*-Actin served as a loading control. *P<0.05 *versus* ctrl; **P<0.05 *versus* Tm, *n*=5. (**c**) Western blot analysis and quantitative assay for ATF4, p-eIF2*α* and CHOP protein expressions. *β*-Actin served as a loading control. *P<0.05 *versus* ctrl; **P<0.05 *versus* Tm, *n*=5. (**d**) Expression of CHOP by immunofluorescence (Green). Nuclei were counterstained with DAPI; *n*=5 for each group

From the above data, we can see that VSOR Cl^−^ currents from the tunicamycin group activate more than that of the control. However, molecular identity of VSOR Cl^−^ channel remains unknown. To test whether VSOR Cl^−^ channel mediates tunicamycin-induced ER stress of cardiomyocytes, we treated cells with DIDS, a non-selective Cl^−^ channel blocker, and DCPIB, a selective VSOR Cl^−^ channel blocker. Our study and others proved that ER stress-related proteins such as GRP78, p-eIF2*α* and ATF4 reached their peak after 100 ng/ml tunicamycin exposure for 24 h (data not shown). DIDS and DCPIB application significantly suppressed tunicamycin-induced upregulation of chaperone protein GRP78 and the transcriptional factor ATF4 (Figures 2a and c), and downregulation of XBP1S (Figure 2b). DIDS and DCPIB also attenuated the phosphorylation of eIF2*α* occurring in tunicamycin-triggered ER stress (Figure 2c). Furthermore, DIDS and DCPIB not only decreased the cellular level of the C/EBP homologous protein (CHOP), but also inhibited the nuclear translocation of CHOP (Figures 2c and d). Therefore, suppression of VSOR Cl^−^ channel by DIDS and DCPIB impedes tunicamycin-induced ER stress of cardiomyocytes.

**Figure 3:**
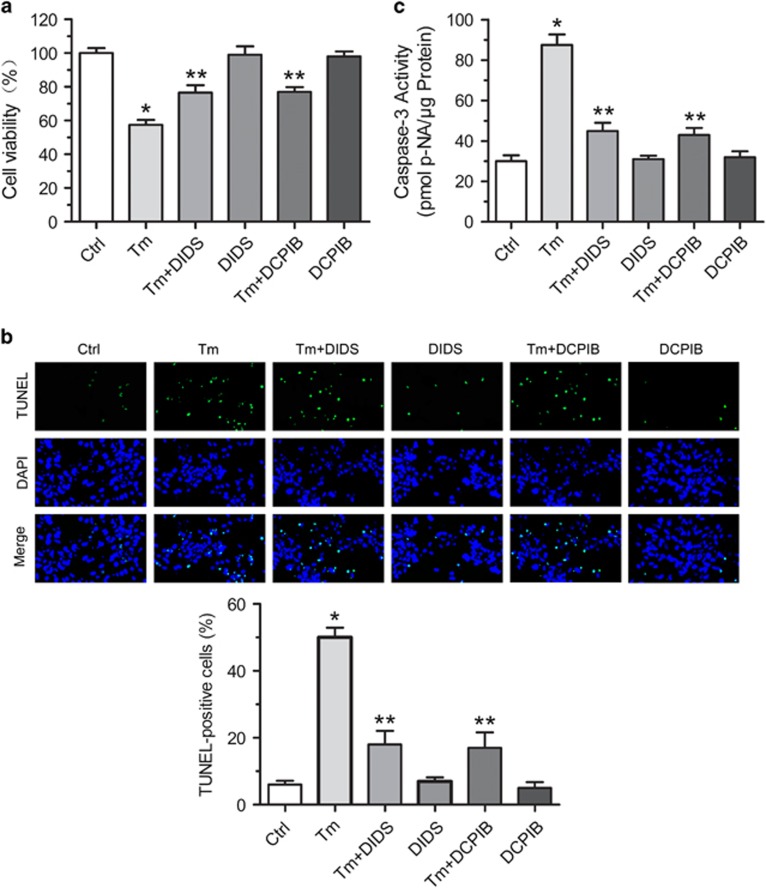
Protective effect of VSOR Cl^−^ channel inhibitors on tunicamycin-induced cardiomyocyte death. Cardiomyocytes were treated with Tm (100 ng/ml) in the presence or absence of DIDS or DCPIB for 72 h. (**a**) Protective effect of DIDS and DCPIB on Tm-induced cardiomyocyte viability measured by MTT-assay, respecitvely. *P<0.05 *versus* ctrl; **P<0.05 *versus* Tm, *n*=5. (**b**) TUNEL staining of cardiomyocytes. Quantitative analysis of TUNEL-positive cardiomyocytes after Tm treatment with or without DIDS (75 *μ*M) and DCPIB (2 *μ*M) for 72 h. *P<0.05 *versus* ctrl; **P<0.05 *versus* Tm, *n*=5. (**c**) Activity analysis of caspase-3. *P<0.05 *versus* ctrl; **P<0.05 *versus* Tm, *n*=5

We next probed the role of VSOR Cl^−^ channel in ER stress-induced apoptosis of cardiomyocytes. DIDS and DCPIB treatment protected cardiomyocytes from tunicamycin-induced impairment of cell viability (Figure 3a). Consistently, tunicamycin-elicited apoptosis of cardiomyocytes was inhibited by DIDS and DCPIB, as revealed by TUNEL staining and activation detection of capase-3 (Figures 3b and c).

### VSOR Cl^−^ channel blockers protect cardiomyocytes through the CHOP–Wnt pathway

**Figure 4:**
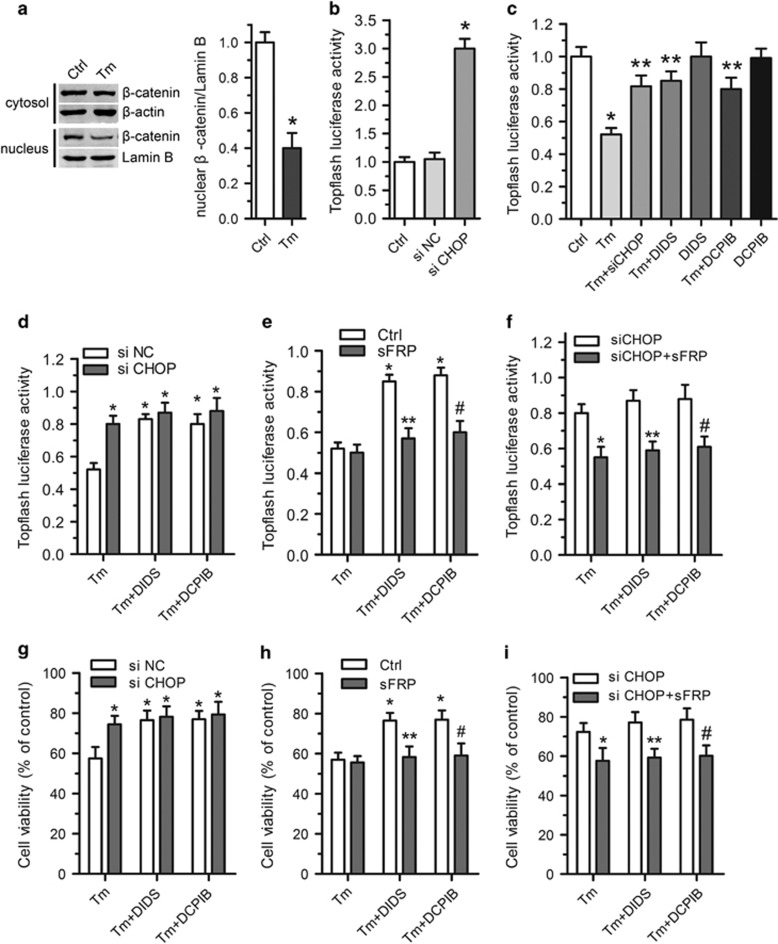
The CHOP-Wnt pathway in VSOR Cl^−^ channel blockers mediated protective role. (**a**) Western blot analysis and quantitative assay of both cytosol and nuclear expression of *β*-catenin in cardiomyocytes after Tm treatment with or without DIDS (75 *μ*M) and DCPIB (2 *μ*M) for 24 h; *n*=5 for each group. (**b**) Topflash and pTK-Rennila luciferase vector were transfected with siNC or siCHOP for 36 h in H9C2 cells. *P<0.05 *versus* ctrl, *n*=5. (**c**) H9C2 cells were transfected with Topflash and pTK-Rennila luciferase vector with or without siCHOP. After 12 h transfection, cells were additionally treated with tunicamycin with or without DIDS, DCPIB for 24 h, respectively. *P<0.05 *versus* ctrl; **P<0.05 *versus* Tm, *n*=5. (**d**–**f**) Topflash and pTK-Rennila luciferase vector with or without siCHOP were transfected for 12 h in H9C2 cells. After 12 h transfection, cells were additionally treated as indicated for 24 h, respectively. (**d**) *P<0.05 *versus* Tm+siNC, *n*=5; (**e**) *P<0.05 *versus* Tm; **P<0.05 *versus* Tm+DIDS; ^#^*P*<0.05 *versus* Tm+DCPIB, *n*=5; (**f**) *P<0.05 *versus* Tm+siCHOP; **P<0.05 *versus* Tm+siCHOP+DIDS; ^#^*P*<0.05 *versus* Tm+siCHOP+DCPIB, *n*=5. (**g**–**i**) Cardiomyocytes were transfected with or without siCHOP for 24 h, and then cells were additionally treated as indicated for 72 h. Cell viability was measured by the MTT-assay in cardiomyocytes. (**g**) *P<0.05 *versus* Tm+siNC, *n*=5; (**h**) *P<0.05 *versus* Tm; **P<0.05 *versus* Tm+DIDS; ^#^*P*<0.05 *versus* Tm+DCPIB, *n*=5; (**i**) *P<0.05 *versus* Tm+siCHOP; **P<0.05 *versus* Tm+siCHOP+DIDS; ^#^*P*<0.05 *versus* Tm+siCHOP+DCPIB, *n*=5

To explore the involvement of Wnt on tunicamycin-induced cardiomyocyte injury, *β*-catenin was examined in cardiomyocytes. Our data showed that tunicamycin significantly decreased the nuclear localization of *β*-catenin (Figure 4a). In addition, tunicamycin could also decrease Topflash activity (Figure 4c). Moreover, Topflash activity reversely correlated with CHOP (Figure 4b). Interestingly, siCHOP prevented the downregulation of Topflash activity induced by tunicamycin (Figures 4c and d), suggesting that Wnt is a CHOP target *in vitro*.

Treatment with DIDS and DCPIB markedly increased Topflash activity compared with the tunicamycin group (Figure 4c), whereas siCHOP and DIDS, DCPIB combination had no extra effect on Topflash activity, compared with siCHOP alone (Figure 4d). Taken together, VSOR Cl^−^ channel blockers invert tunicamycin-induced Wnt expression through CHOP-dependent regulation.

Next, we examined the cell viability. siCHOP or treatment with DIDS and DCPIB improved the cell survival (Figure 4g), whereas siCHOP and VSOR Cl^−^ channel blockers DIDS and DCPIB together had no extra effect on cell viability induced by tunicamycin. Taken together, VSOR Cl^−^ channel blockers protect cardiomyocytes from tunicamycin-induced apoptosis through CHOP. To investigate the role of Wnt in VSOR Cl^−^ channel blockers-induced protective effect, sFRP was used to inhibit Wnt. sFRP itself had no obvious effect on cell apoptosis (data not shown). In contrast, the Wnt activition by DIDS and DCPIB was nearly fully reversed by sFRP (Figure 4e), and the anti-apoptotic effect of DIDS and DCPIB was nearly fully blunted by sFRP (Figure 4h). Taken together, VSOR Cl^−^ channel blockers protect cardiomyocytes from tunicamycin-induced apoptosis through regulation of Wnt.

To determine the functional significance of the CHOP-Wnt pathway, siCHOP and sFRP were used to inhibit CHOP and Wnt, respectively. The Wnt activition effect of siCHOP and/or VSOR Cl^−^ channel blockers was nearly fully reversed by sFRP (Figure 4f), and the anti-apoptotic effect of siCHOP and/or VSOR Cl^−^ channel blockers was nearly inhibited by sFRP (Figure 4i). Taken together, these results support the concept that VSOR Cl^−^ channel blockers protect cardiomyocytes from tunicamycin-induced apoptosis through CHOP-dependent regulation of Wnt expression.

### Increased ROS mediate VSOR Cl^−^ currents in tunicamycin-exposed cardiomyocytes

**Figure 5:**
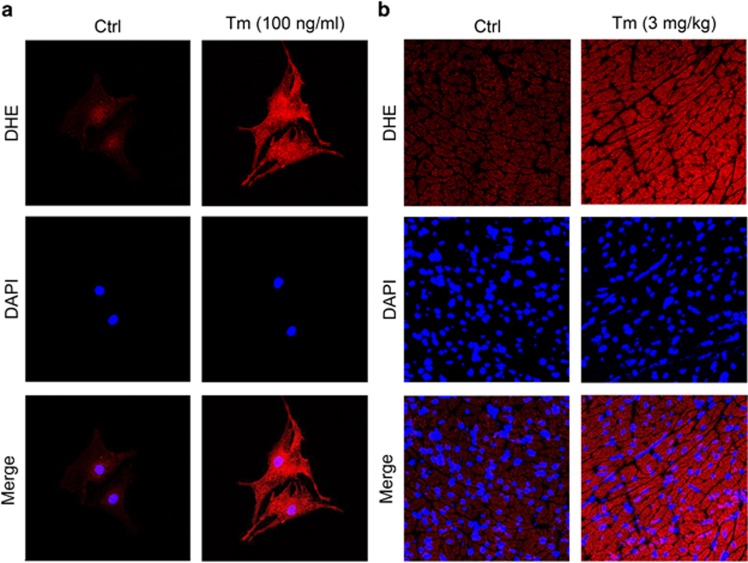
Tunicamycin induced ROS production *in vitro* and *in vivo*. (**a**) Effect of Tm (100 ng/ml) for 48 h on the ROS level of cardiomyocytes monitored by dihydroethidine (DHE) staining (Red). Nuclei were counterstained with DAPI; *n*=5 for each group. (**b**) Increased ROS accumulation in tunicamycin (3 mg/kg, 48 h, i.p.)-exposed myocardium was revealed by DHE staining (Red). Nuclei were counterstained with DAPI; *n*=5 for each group

To test whether ROS participate in the activation process of VSOR Cl^−^ channel in responses to tunicamycin, superoxide anion (O_2_^−^) was evaluated by DHE staining. Compared with control group, O_2_^−^ was significantly increased in cultured cardiomyocytes after 48 h exposure to tunicamycin (Figure 5a). Similarly, tunicamycin treatment of mice (3 mg/kg, i.p.) for 48 h increased intracellular O_2_^−^ of hearts (Figure 5b). Thus, apparent ROS production occurs concomitantly with tunicamycin-induced ER stress.

**Figure 6:**
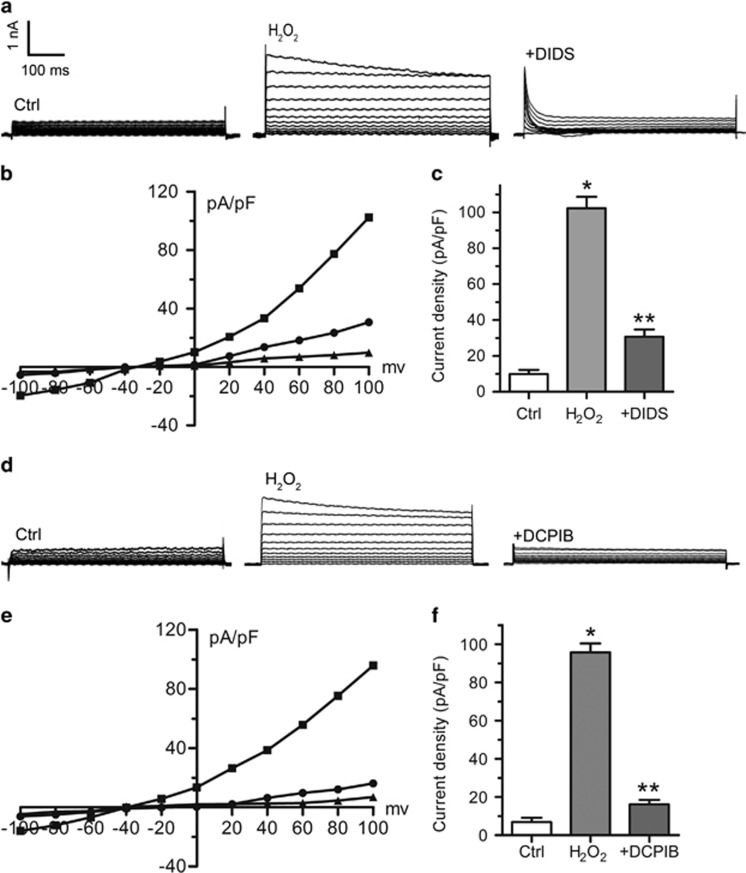
ROS induce VSOR Cl^−^ currents in cardiomyocytes. (**a**) Background Cl^−^ currents recorded under isosmotic solution (Ctrl). H_2_O_2_ (500 *μ*M)-induced Cl^−^ currents exhibiting phenotypic properties of I_Cl,Vol_ (H_2_O_2_). H_2_O_2_-induced VSOR Cl^−^ currents were inhibited by adding DIDS (500 *μ*M); *n*=5 for each group. (**b**) Corresponding current-voltage (I-V) relationship for the mean current densities of isosmotic (▴), H_2_O_2_ (▪) and H_2_O_2_ with DIDS (●) conditions. (**c**) Current densities at +100 mV from B. *P<0.05 *versus* Ctrl; **P<0.05 *versus* H_2_O_2_, *n*=5. (**d**) Negligible background Cl^−^ currents recorded under isosmotic solution (Ctrl). H_2_O_2_ (500 *μ*M)-induced Cl^−^ currents exhibiting representative properties of VSOR Cl^−^ currents (H_2_O_2_). H_2_O_2_-induced VSOR Cl^−^ currents were inhibited by adding DCPIB (10 *μ*M). *n*=5 for each group. (**e**) Corresponding current-voltage (I-V) relationship for the mean current densities of Ctrl (▴), Tm (▪) and Tm with DIDS (●) conditions. (**f**) Current densities at +100 mV from (**e**). *P<0.05 *versus* Ctrl; **P<0.05 *versus* H_2_O_2_, *n*=5

**Figure 7:**
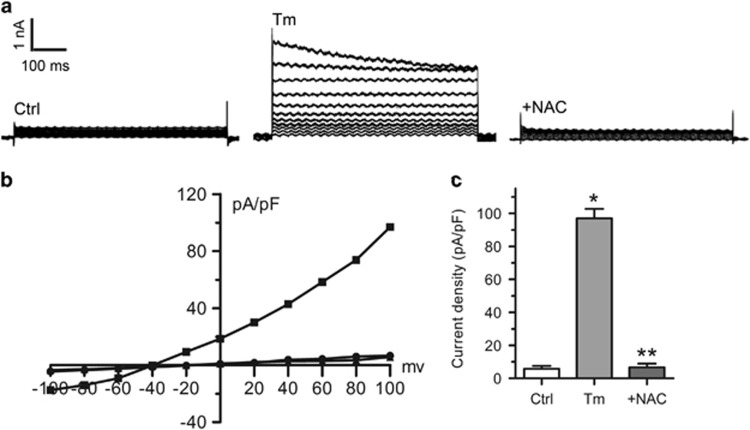
ROS production mediates tunicamycin induced VSOR Cl^−^ currents. (**a**) Background Cl^−^ currents recorded under isosmotic solution (Ctrl). Tm (3 *μ*g/ml)-induced VSOR Cl^−^ currents exhibiting phenotypic properties of I_Cl,Vol_ (Tm). Tm-induced VSOR Cl^−^ currents were inhibited by the ROS scavenger NAC (10 mM). n=5 for each group. (**b**) Corresponding current-voltage (I-V) relationship for the mean current densities of isosmotic (▴), Tm (▪) and Tm with NAC (●) conditions. (**c**) Current densities at +100 mV from (**b**). *P<0.05 *versus* Ctrl; **P<0.05 *versus* Tm, *n*=5

Next, we explored whether ROS mediate VSOR Cl^−^ currents in tunicamycin-exposed cardiomyocytes. Hydrogen peroxide (H_2_O_2_) and ROS scavenger NAC were used to mimic ROS and block ROS production in cardiomyocytes, respectively. Extracellular application of H_2_O_2_ (500 *μ*M, 7–10 min) directly elicited VSOR Cl^−^ currents, which could be inhibited by DIDS (500 *μ*M, 5–7 min, 77.49±3.11%, *n*=5, *P*<0.05) and DCPIB (10 *μ*M, 6-7 min, 88.74±4.86%, *n*=5, *P*<0.05) (Figure 6). In addition, tunicamycin-induced activation of VSOR Cl^−^ currents was almost completely abolished through scavenging ROS with NAC (10 mM, 5–10 min, 99.07±5.86% *n*=5, *P*<0.05) (Figure 7). Therefore, ROS production is pivotally involved in VSOR Cl^−^ channel activation.

### VSOR Cl^−^ channel blockers *in vivo* counteract ER stress-triggered cardiomyocyte apoptosis and improve cardiac function

**Figure 8:**
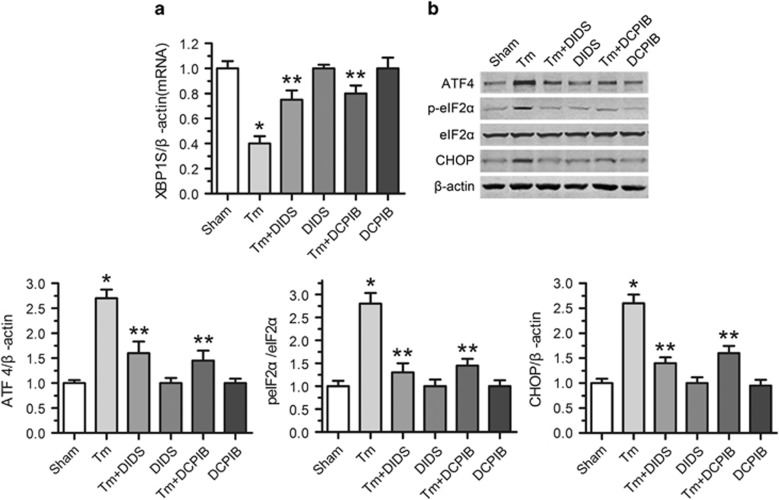
VSOR Cl^−^ channel blockers rescue tunicamycin-induced ER stress *in vivo*. VSOR Cl^−^ channel was blocked with DIDS and DCPIB for 24 h before assessment of ER stress. (**a**) qRT-PCR assay for XBP1S expression. *β*-actin served as a loading control. *P<0.05 *versus* Sham; **P<0.05 *versus* Tm, *n*=5. (**b**) Western blot analysis and quantitative assay for ATF4, p-eIF2*α* and CHOP protein expressions. *β*-actin served as a loading control. *P<0.05 *versus* Sham; **P<0.05 *versus* Tm, *n*=5

**Figure 9:**
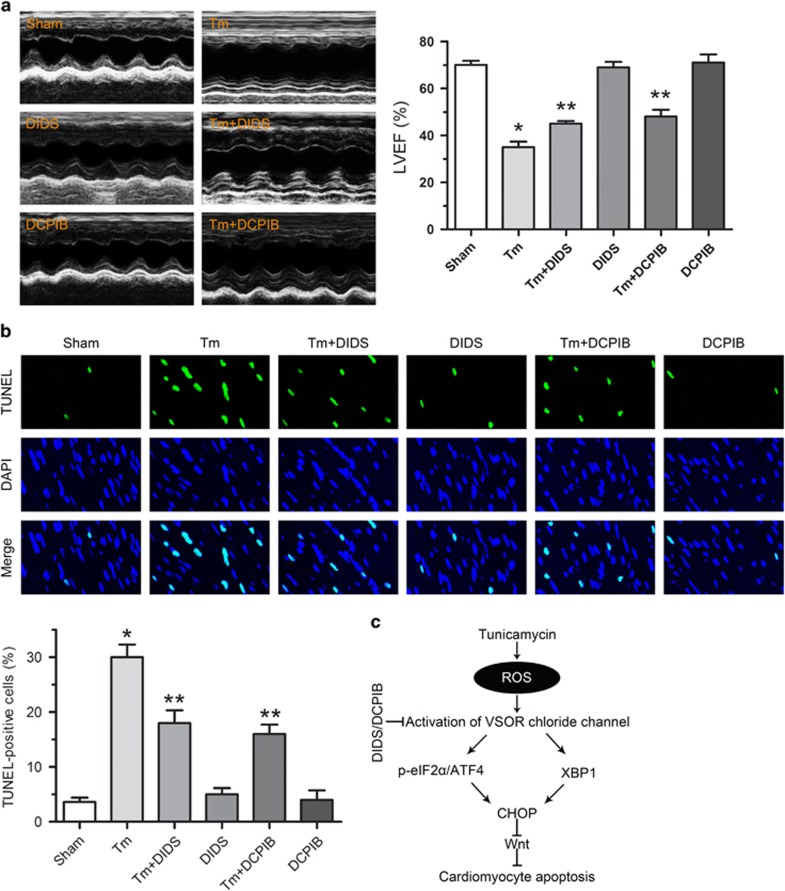
VSOR Cl^−^ channel blockers improve cardiac function and attenuate tunicamycin-induced cardiomyocyte apoptosis. VSOR Cl^−^ channel was blocked with DIDS and DCPIB for 48 h before assessment of cardiac function and apoptosis, respectively. (**a**) Echocardiographic assessment of the left ventricular ejection fraction (LVEF). *P<0.05 *versus* Sham; **P<0.05 *versus* Tm, *n*=5. (**b**) TUNEL staining of apoptotic cells in myocardium, and quantified data displaying % of apoptosis. *P<0.05 *versus* Sham; **P<0.05 *versus* Tm, *n*=5. (**c**) Schematic representation of how VSOR Cl^−^ channel involved in tunicamycin-induced ER stress. Tunicamycin results in increased ROS production in the heart, which in turn activates VSOR Cl^−^ currents. VSOR Cl^−^ currents lead to increased ER stress, resulting in increased cell apoptosis and cardiac contractile dysfunction through CHOP-dependent regulation of Wnt expression.

To evaluate the effects of ER stress on cardiac contractile function *in vivo*, mice were administered with tunicamycin (3 mg/kg, i.p. for 48 h), and left ventricular eject fraction (LVEF) was measured by echocardiography. Tunicamycin challenge did not significantly affect the body or organ (the lung, liver or kidney) weight, or the heart size (heart-to-body weight ratio) and the heart rate. In addition, LV wall thickness and ventricular septal thickness were comparable among all groups (Data not shown). In contrast, tunicamycin challenges significantly increased p-eIF2*α*, ATF4 and CHOP, and decreased XBP1S, LVEF, suggesting that tunicamycin led to ER stress-induced cardiac contractile dysfunction (Figures 8 and 9a). Given that ER stress is known to elicit myocardial damage through apoptotic cell death, we next examined the effect of tunicamycin on apoptosis of cardiomyocytes. TUNEL assay revealed that tunicamycin induced apparent apoptosis (Figure 9b). Therefore, tunicamycin triggers serious apoptosis of cardiomyocytes, providing an effective model for studies on ER stress-induced cardiac contractile dysfunction (CCD).

To investigate whether blocking VSOR Cl^−^ channel could improve ER stress-triggered cardiac contractile dysfunction, DIDS and DCPIB were used to block VSOR Cl^−^ channel in tunicamycin-administered mice, respectively. As a result, DIDS and DCPIB treatment significantly increased XBP1S and decreased p-eIF2*α*, ATF4, CHOP, TUNEL-stained cell numbers, suggesting an inhibition of tunicamycin-induced apoptosis of cardiomyocytes by VSOR Cl^−^ channel blockers (Figures 8 and 9b). Echocardiography showed that DIDS and DCPIB mitigated the cardiac function impairment caused by tunicamycin, respectively (Figure 9a). Therefore, blockade of VSOR Cl^−^ channel protects cardiomyocytes from ER stress-induced apoptosis or loss of contractility.

## Discussion

CCD represents the common clinical manifestation of a class of cardiovascular disorders. Given that the etiologic conditions of CCD, for example, atherosclerosis, ischemia/reperfusion injury of the heart, alcoholic cardiomyopathy, autoimmune cardiomyopathy, myocardial infarction, cardiac hypertrophy and heart failure frequently dampen protein folding or cause calcium depletion in the ER,^36, 37, 38, 39^ the essential role of ER stress in CCD has began to be unveiled. Consistent with accumulating studies demonstrating the critical involvement of ER stress under various cardiac pathological states,^16, 36, 40^ we found that tunicamycin-induced ER stress promoted apoptosis and severely impaired the cardiac contractility. ER stress inducer tunicamycin caused excessive generation of ROS, which consequently activated VSOR Cl^−^ channel and ultimately accelerated ER stress, and thus apoptosis of cardiomyocytes by inhibition of Wnt activation (Figure 9c). The newly unraveled ROS- and VSOR-dependent pathways are critically involved in ER stress-related cardiac contractile dysfunction in a mouse CCD model.

Despite the defined roles of ER stress-related apoptosis of cardiomyocytes in cardiovascular diseases such as myocardial infarction, ischemia-reperfusion injury, cardiomyopathy, atherosclerosis and heart failure, it remains largely uncharacterized how these abnormalities activate the molecular machineries of ER stress and related apoptosis of cardiac myocytes. However, accumulating data have suggested the production of ROS under the aforementioned pathological conditions.^41, 42, 43^ Given the established role of ROS in inducing ER stress, we propose that in addition to triggering ER stress via disruption of protein *N*-glycosylation and proper folding, tunicamycin also propagates ER stress by facilitating excessive ROS generation, which is supported by both our findings and previous reports that tunicamycin treatment resulted in a profound increase in intracellular ROS.^44, 45^ ROS have been well-documented in mediating cell signals leading to organelle damage, tissue and organ degeneration, and aging.^46^ The production of ROS was found to correlate closely with myocardial infarction, ischemia-reperfusion injury, cardiomyopathy, atherosclerosis and heart failure.^47, 48^ ROS is also involved in other cellular processes, for example, the activation of ion channels.^49^ Presumably due to the multifaceted roles of ROS in stress signaling, we established that tunicamycin treatment of cardiomyocytes leads to extensive activation of the canonical signaling of the ER stress, mainly the eIF2*α*/ATF4 and XBP1S pathways. Therefore, tunicamycin-elicited ER stress may appropriately mimick the complicated environmental stimuli responsible for the development of cardiovascular diseases.

Apoptosis induction requires persistent cell shrinkage, that is, AVD, reminiscent of the role of VSOR Cl^−^ channel in the outward rectifying Cl^−^ currents in cardiomyocytes.^24^ Blockade of chloride channel by DIDS and DCPIB could prevent cardiomyocyte apoptosis.^31, 50^ The volume-sensitive chloride channel inhibitors IAA-94 and DIDS prevent both contractile dysfunction and apoptosis induced by doxorubicin through PI3K, Akt and Erk 1/2.^51^ Although VSOR Cl^−^ channel participated in apoptosis via the mitochondrial or death receptor pathway, whether VSOR Cl^−^ channel-induced AVD participates in ER stress is still unknown. Our data revealed that tunicamycin increased the densities of VSOR Cl^−^ currents, which displayed voltage-dependent inactivation at large positive voltages. The tunicamycin-induced activation of VSOR Cl^−^ channel was inhibited by adding DIDS, a non-selective Cl^−^ channel blocker. To distinguish from other possible styles of Cl^−^ currents, DCPIB, a selective inhibitor of VSOR Cl^−^ channel, was selected and our data revealed that DCPIB effectively inhibits tunicamycin-induced VSOR Cl^−^ currents. DIDS and DCPIB decreased ER stress signaling and reduced apoptosis of cardiomyocytes and mitigated CCD in animals, respectively. Albeit principally activated by osmotic cell swelling,^52, 53, 54^ VSOR Cl^−^ channel has recently been found activated by ROS eliciting cell swelling.^29, 55, 56, 57, 58^ In agreement with these reports, we established here that extracellular application of H_2_O_2_ activated VSOR Cl^−^ currents, which was blocked by DIDS and DCPIB, respectively. Moreover, tunicamycin-induced activation of VSOR Cl^−^ currents was almost completely eliminated by the antioxidant NAC. These data indicate that ROS are required for tunicamycin-induced VSOR activation. However, further investigations are necessary to dissect the molecular link between VSOR activation and ER stress. Given the characterized role of calcium channel in mediating cell responses to osmotic changes,^59^ it is worth further investigation whether VSOR elicits ER stress via cell volume regulation and subsequently disruption of Ca^2+^ homeostasis.

Next, we demonstrated that DIDS and DCPIB significantly decrease tunicamycin-induced CHOP upregulation, respectively. These results suggest that DIDS and DCPIB attenuate tunicamycin-induced ER stress through the CHOP pathway. However, the precise downstream of CHOP remains to be further elucidated.

Wnt is an essential cell protective mediator under multiple conditions. Wnt is an important effector in the anti-apoptotic signaling. Previous study revealed that increased Wnt signal by dishevelled-1 knockdown attenuates cyclosporine A-induced apoptosis in H9C2 cardiomyoblast cells.^33^ However, whether Wnt participates in ER stress-induced cardiomyocyte apoptosis has not been reported. In our study, tunicamycin downregulated Wnt activity, while siCHOP inverted downregulation of Wnt induced by tunicamycin. These indicate that Wnt is a CHOP-regulated gene in cardiomyocytes. Treatment with VSOR Cl^−^ channel blockers significantly increase Wnt activity; however, siCHOP and VSOR Cl^−^ channel blockers combination have no extra effects on Wnt. These results demonstrate that VSOR Cl^−^ channel blockers reverse tunicamycin-induced Wnt through the CHOP–dependent pathway.

Autophagy is an essential survival mechanism during energy stress in the heart.^60^ Moreover, autophagy has cytoprotective role during ER stress and can delay or prevent UPR-dependent apoptosis activation to relieve the stress.^11^ Next, we will try to elucidate the relationship of VSOR chloride channel, autophagy and ER stress.

Our study provides strong evidence that tunicamycin results in excessive generation of ROS in the heart, which in turn activates VSOR Cl^−^ currents. Increased VSOR Cl^−^ currents lead to ER stress, resulting in increased cell apoptosis and cardiac contractile dysfunction through CHOP-dependent regulation of Wnt expression, demonstrating the feasibility for future treatment of CCDs with VSOR Cl^−^ channel blockers.

## Materials and Methods

### Reagents and antibodies

Tunicamycin, 4,4′-Diisothiocyanatostilbene-2,2′-disulfonic acid disodium salt hydrate (DIDS), 4-(2-butyl-6,7-dichlor-2-cyclopentyl- indan-1-on-5-yl)oxybutyric acid (DCPIB), 5-bromo-2-deoxyuridine (BrdU), 3-(4,5- dimethylthiazol-2-yl)-2, 5-diphenyltetrazolium bromide (MTT), N-acetyl-l-cysteine (NAC) and antibody for *β*-actin were purchased from Sigma-Aldrich Corporation (St. Louis, MO, USA). sFRP was purchased from R&D Systems (Minneapolis, MN, USA). Antibody against GRP78 was from Bioworld Technology (St. Louis Park, MN, USA). Antibodies against CHOP and ATF4 were obtained from Santa Cruz Biotechnology (Santa Cruz, CA, USA). Phospho-eIF2*α*, total eIF2*α*, *β*-catenin and *α*-actinin were purchased from Cell Signaling Technology (Danvers, MA, USA). Antibody against Lamin B was from Boster (Wuhan, China). Dihydroethidium (DHE) was from Molecular Probes (Eugene, OR, USA). Terminal Deoxynucleotidyltransferase-mediated dUTP Nick End Labeling (TUNEL) was from Roche Applied Science (Sandhofer Strasse, Mannheim, Deutschland).

### *In vivo* model of ER stress with tunicamycin i.p. injection

All procedures were in accordance with the Guide for the Care and Use of Laboratory Animals published by the US National Institutes of Health (NIH Publication No. 85-23, revised 1996) and approved by the Fourth Military Medical University Committee on Animal Care. To trigger ER stress *in vivo*, mice were given an i.p. injection of tunicamycin, an inhibitor of *N*-glycosylation in the ER (3 mg/kg), for 48 h before assessment of mechanical, morphological and biochemical features as described previously.^10^ Control mice were given similar amount of saline (i.p.). Animals were killed by intraperitoneal injection of ketamine (200 mg/kg) and xylazine (100 mg/kg), and hearts were rapidly excised. Then, the hearts were harvested, and frozen sections were prepared. TUNEL staining was performed using the *in situ* TUNEL cell death detection kit (Roche Applied Science), followed by nuclear counterstaining with DAPI.

### Primary culture of cardiomyocytes and induction of ER stress *in vitro*

Cardiomyocytes were prepared from newborn Sprague–Dawley rats as described previously.^30^ Neonatal rats were killed by decapitation. In brief, neonatal rat ventricles were enzymatically digested, and cardiomyocytes were purified through 1 h incubation. Cardiomyocytes were cultured in DMEM medium supplemented with 10% fetal bovine serum and 100 *μ*M BrdU for 16–24 h. To elicit ER stress *in vitro*, isolated cardiomyocytes were incubated with tunicamycin (100 ng/ml) at 37 °C.

### Echocardiography to assess cardiac function

Cardiac function was measured using echocardiography (VisualSonics VeVo 770) 48 h after tunicamycin (3 mg/kg) i.p. injection. Mice were anesthetized with isoflurane (2.5%, 10 *μ*l/g). Cardiac geometry and function were evaluated using 2-D guided M-mode echocardiography equipped with a 15-16 MHz linear transducer. Left ventricular (LV) anterior and posterior wall dimensions during diastole and systole were recorded from three consecutive cycles in M-mode using methods adopted by the American Society of Echocardiography. LVEF (left ventricular eject fraction) was calculated from LV end-diastolic (LVEDD) and end-systolic (LVESD) diameters using the equation [(LVEDD^3^−LVESD^3^) /LVEDD^3^] × 100%.

### Determination of cell viability

Cell viability was assessed by MTT assay. In brief, cardiomyocytes were plated into 96-well culture plates at a density of 5 × 10^4^ /well (100 *μ*l). MTT was added into each well with a final concentration of 0.5 mg/ml, and cells were incubated for 4 h at 37 °C. The formazan crystals were dissolved in dimethyl sulfoxide (DMSO, 150 *μ*l/well). The absorbance was detected at 490 nm using a microplate reader (Bio-Rad, Philadelphia, PA, USA).

### Tunel assay

Apoptosis was assayed by TUNEL staining using the *in situ* TUNEL cell death detection kit according to the manufacturer's instructions. In brief, cells were fixed with 4% paraformaldehyde and permeabilized with 0.3% Triton X-100 for 1 h at room temperature, and then washed twice with PBS. Cells were then incubated with the TUNEL assay reaction mixture at 37 °C for 1 h, followed by nuclear counterstaining with DAPI. The number of TUNEL-positive cells in each field was counted and expressed as a percentage of the total number of cells.

### Patch-clamp experiments

**Figure 10:**
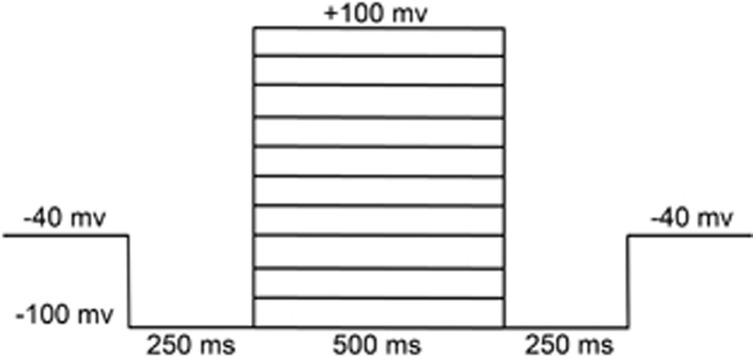
Corresponding step protocol used to elicit current trace. To observe the current–voltage relationships, step pulses were generated from a holding potential of −40 mV to test potentials from −100 to +100 mV with 20 mV increments. To record the greater magnitude of pulse-induced currents, the −100 mv conditioning pulse was applied before and after test potentials.

The VSOR Cl^−^ currents were recorded with an Axon Multiclamp 700B amplifier and Digidata1322A (Axon Instruments, Foster, CA, USA) using the whole-cell configuration. Voltage clamp protocols (Figure 10) and data acquisition were controlled by pClamp10 software. Pipettes were fabricated from borosilicate glass capillaries using a micropipette puller (P-2000, Sutter Instrument, Novato, CA, USA) with resistance of 3–5 MΩ when filled with pipette solution. Liquid junction potentials were calculated with JPCalc in pClamp 10 and corrected on-line. For whole-cell recordings, the capacitative transients and access resistance were maximally compensated. The pipette solution (103 mM CsOH, 103 mM Aspartic acid, 25 mM CsCl, 5 mM Mg-ATP, 0.3 mM Na_3_-GTP, 5 mM EGTA, 10 mM HEPES, and 30 mM mannitol, pH7.4 adjusted with CsOH, 295 mosmol/Kg H_2_O) was used to selectively record whole-cell Cl^−^ currents. The isotonic bathing solution contained 85 mM N-methyl-D-glucamine (NMDG), 85 mM HCl, 10 mM NaCl, 2 mM 4-aminopyridine (4-AP), 2.5 mM BaCl_2_, 0.33 mM NaH_2_PO_4_, 4 mM MgCl_2_, 5 mM Tetraethylammonium-Cl (TEA-Cl), 10 mM HEPES, 5.5 mM glucose and 85 mM mannitol (pH7.4 adjusted with NMDG-OH, 305 mosmol/Kg H_2_O). Tetrodotoxin (TTX, 8 *μ*M) and nifidipine (5 *μ*M) were routinely included in bath solutions to block Na^+^ channel and L-type Ca^2+^ channel, respectively. The osmolality of all solutions was measured using a freezing-point depression osmometer (OM802, Vogel, Giessen, Germany).

### Immunofluorescence for expression of GRP78 and CHOP in cardiomyocytes

Cardiomyocytes were incubated with indicated doses of drugs. Next, cells were fixed with 4% paraformaldehyde for 10 min and permeabilized with 0.3% Triton X-100 for 1 h at room temperature. Immunofluorescence assessment of cardiomyocyte expression of GRP78 was carried out using cardiomyocyte-specific mouse monoclonal anti-*α*-actinin (1 : 100 in antibody dilution) and rabbit polyclonal anti-GRP78 (1 : 100 in antibody dilution), followed by staining with goat anti-mouse secondary Flour-594 antibody (1 : 200 in antibody dilution; Invitrogen, Carlsbad, CA, USA) and goat anti-rabbit secondary Alexa Flour 488 (1 : 200 in antibody dilution; Invitrogen). Cardiomyocyte expression of CHOP was carried out using mouse monoclonal anti-*α*-actinin (1 : 100 in antibody dilution) and rabbit polyclonal anti-CHOP (1 : 100 in antibody dilution), followed by staining with goat anti-mouse secondary Flour-594 antibody (1 : 200 in antibody dilution; Invitrogen) and goat anti-rabbit secondary Alexa Flour 488 (1 : 200 in antibody dilution; Invitrogen). The sections were observed and images were captured by confocal laser scanning microscopy (Nikon, Tokyo, Japan).

### H9C2 cell culture and RNA interference

H9C2 cells, a cell line derived from fetal rat heart, were purchased from American Type Culture Collection (ATCC, USA) for the siRNA and repoter assay study due to the poor transfection efficacy of primary cultured rat cardiomyocytes. Cells were cultured in DMEM medium containing 10% fetal bovine serum (FBS) in a 5% CO_2_ incubator at 37 °C.

Small interfering RNA against CHOP was performed as described previously.^61^ CHOP–specific short interfering RNA (siCHOP) was obtained from GenePharma (Shanghai, China). Target sequence of siCHOP was 5′-CGAAGAGGAAGAAUCAA- A-3′, and the negative control sequence was 5′-UUCUCCGAACGUGUCACGU- TT-3′.

### Reporter assay

Topflash and internal control pRL-TK vectors were co-transfected into H9C2 cells. After transfection for 36 h, H9C2 cells were lysed using passive lysis buffer. Firefly and Renilla luciferase activities were analyzed using the dual-luciferase reagent assay kit (Promega, USA) according to the manufacturer's instructions.

### Western blot analysis

Cardiomyocytes were lysed by RIPA containing a protease inhibitor cocktail. Electrophoresis and immunobloting were done as described previously.^62^ For the densitometric analysis, optical density was measured on the inverted digital images using Image J software.

### Quantitative real-time PCR

cDNA synthesis was performed with QuantiTect Reverse Transcription Kit (TaKaRa Biotech, Dalian, China). PCR was performed on an ABI prism 7500 with the Power SYBR Green PCR Master Mix (TaKaRa Biotech, China). *β*-actin was used as an endogenous control to normalize the amount of RNA. XBP1S forward: 5'-GCTTGTGATTGAGAACCAGG-3' and reverse: 5'-GGCCTGCACCTGCTGCGGACTC-3' *β*-actin forward: 5'-AGAGGGAAATCGTGCGTGAC-3' and reverse: 5'-TTCTCCAGGGAGGAAGAGGAT-3'.

### Intracellular fluorescence measurement of O_2_^−^

Intracellular superoxide was monitored by changes in fluorescence intensity resulting from intracellular probe oxidation according to the previously described method.^63^ Cardiomyocytes were loaded with 5 *μ*M dihydroethidium (DHE) for 30 min at 37 °C and washed twice with PBS buffer. Cells were captured using a confocal microscope (Nikon).

### Statistical analysis

Results were presented as mean±S.E.M. All data were subjected to ANOVA, followed by Bonferroni correction for *post-test*. *P*<0.05 was considered statistically significant.

## Abbreviations

ERS: endoplasmic reticulum stress
CCD: cardiac contractile dysfunction
VSOR: volume-sensitive outwardly rectifying
DIDS: 4,4'-diisothiocya-natostilbene-2,2'-disulfonicacid
DCPIB: 4-(2-butyl-6,7-dichlor-2-cyclopentyl-indan-1-on-5-yl) oxybutyric acid
GRP78: glucose-regulated protein78kD
ROS: reactive oxygen species
CHOP: C/EBP homologous protein
Tm: tunicamycin
AVD: apoptotic volume decrease
